# PERCH in Perspective: What Can It Teach Us About Pneumonia Etiology in Children?

**DOI:** 10.1093/cid/cix075

**Published:** 2017-05-27

**Authors:** Keith P. Klugman, Gail L. Rodgers

**Affiliations:** 1 Bill & Melinda Gates Foundation, Seattle, Washington

**Keywords:** pneumonia, diagnosis, bacterial, viral; PERCH.

## Abstract

The pneumonia team at the Bill & Melinda Gates Foundation congratulates the Pneumonia Etiology Research for Child Health (PERCH) study on delivering on their grant to collect high-quality data from thousands of children with World Health Organization–defined severe and very severe pneumonia and from controls in 9 diverse sites in 7 low- and middle-income countries. This supplement sets the foundation to understanding this complex study by providing an in-depth description of the study methodology, including discussion of key aspects such as antibiotic pretreatment, chest radiograph interpretation, utility of induced sputum in children, measurement of pathogen density, and use of C-reactive protein, and how these affect pneumonia etiology.

The premise under which the Pneumonia Etiology Research for Child Health (PERCH) study was funded by the Bill & Melinda Gates Foundation deserves consideration [1]. In 2008, around the time PERCH was initiated, pneumonia was the leading cause of death in children <5 years of age, estimated to cause 1.575 million deaths in children <5 years of age [1]. Although pneumonia deaths have decreased significantly, likely due to access to healthcare and to life-saving antibiotics, as well as increased use of *Haemophilus influenzae* type b (Hib) vaccine and pneumococcal conjugate vaccine (PCV) in the poorest countries, it remains the leading killer of children <5 years of age, with an estimated 900000 deaths per year in 2013 [2, 3]. PERCH was designed to shed light on the potentially changing etiology of childhood pneumonia and its mortality, given the rollout of these interventions.

A large part of the funding premise was that obtaining a comprehensive set of specimens from cases and controls, applying state-of-the art laboratory tests, including use of a new, multiplex real-time polymerase chain reaction assay targeting an extensive array of >30 potential pathogens, and standardizing methodologies across a range of epidemiologic and geographic settings in low- and low- to middle-income countries, would update our understanding of pneumonia etiology and mortality [1, 4].

Many of these tests, particularly the molecular technologies, were not available to a previous large study conducted 30 years ago [5]. The US Board of Science and Technology for International Development (BOSTID) study [5]was set up in response to >4 million deaths in children <5 years of age annually attributable to acute respiratory infections (ARIs) during the 1980s. BOSTID used culture and immunofluorescence, in the absence of controls, and found that respiratory syncytial virus (RSV) was the most frequent pathogen identified in nasopharyngeal swabs from children with lower respiratory tract infections. The study concluded that the more frequent identification of viruses than bacteria in the nasopharynx of children with these infections meant that viruses were “more frequently responsible than bacteria for episodes of ARI.” Ironically, the BOSTID data came about at the start of 2 decades in which major respiratory bacterial vaccines were developed (Hib and PCVs) and no progress was made in the delivery of respiratory viral vaccines to children beyond measles, the diagnosis of which was excluded from the BOSTID study. The PERCH study allows us to evaluate pneumonia etiology in a time where many of the countries had introduced conjugate vaccines and access to healthcare had improved. It also allows for comparison of etiologies in countries that have introduced these vaccines at the time of the study with those that had not. The lack of a gold standard in the diagnosis of pneumonia in children, establishment of bacterial etiology, and ascertaining etiology from specimens that are not at the site of infection are fundamental problems that PERCH tackles with new technologies and novel analytic methods. Moving away from the site of infection to an easily accessible site, the nasopharynx, even with the most up-to-date molecular methods, unfortunately does not differentiate cases from controls infected with the major bacterial causes of pneumonia. Therefore, nasopharyngeal sampling has not so far helped much in our understanding of bacterial pneumonia etiology in children; although density may be diagnostic of bacterial pneumonia in adults [6], the overlap in density of carriage in cases and controls is too great to be useful in individual children [7]. Further data on bacterial density are presented herein from PERCH [8], as well as data suggesting that viral density is also not predictive of pneumonia etiology compared to controls [9].

We have made enormous progress and are getting closer to determining the etiology of pneumonia, but there are still many unknowns. Future studies based on patterns of gene expression by these bacteria may be of interest, but unfortunately RNA was not collected as part of the study. The role in nonbacteremic pneumonia of non-vaccine-type pneumococci (and perhaps non–type b *H. influenzae*) post-PCV rollout remains to be determined. The use of a serotype-specific pneumococcal assay in children, like the one used to evaluate vaccine impact on pneumonia in adults [10], would greatly advance the diagnosis of pneumococcal pneumonia, but numerous hurdles remain, including the assessment of the assay in children who may have high density carriage, and the lack of monoclonal antibodies in the assay directed at nonvaccine types, which will be more important in the post PCV10/13 era. Urine collected in PERCH is currently being used in the evaluation and adaptation of this type of assay for use in children (Kate O’Brien, personal communication).

PERCH will be helpful in the debate over the definition of pneumonia in children. Careful training in the World Health Organization (WHO) definition of standardized alveolar consolidation pneumonia [11, 12] allows the evaluation of a subgroup of patients in PERCH meeting that definition, who may be more comparable to children meeting that endpoint in PCV randomized trials. The use of the urine assay in such a subgroup of patients who meet the WHO endpoint pneumonia definition in sites that had not yet introduced PCV may allow the assessment of the sensitivity of a urinary assay for the vaccine types in those children.

A great strength of PERCH is the storage of specimens from cases and controls that may be useful for evaluation of newly discovered pathogens, and also the evaluation of biomarkers. The biobank repository that PERCH has created may yet hold more answers for future technologies to unravel further the etiology of childhood pneumonia.

The detection of bacterial pneumonia remains crucial to understanding bacterial pneumonia burden and to improving treatment algorithms. Transcriptional profiling of host genes has been recently shown to be superior to the more established biomarker, procalcitonin, in adults [13], although promising biomarker data differentiating bacterial pneumonia from controls in children are still elusive.

Preliminary data from the South African PCV efficacy trial suggest that, at least in an urban cohort of children, pneumonia mortality in infants is predicted by a low oxygen saturation in arterial blood together with indicators of undernutrition such as low weight for age and refusal of feeds. This Respiratory Index of Severity in Children (RISC) score [14] has been assessed across the PERCH sites and we await with interest their evaluation of the score and other indices of risk for mortality that may come from the study. PERCH will provide information on case fatality ratios, but a limitation of studies based on hospitalized infants, hopefully with access to oxygen and antibiotics, is that the fatality ratios of children without this access cannot be measured. Given the high predictive value of the molecular detection of RSV, a nasopharyngeal study of infants dying in the community in high mortality areas may shed further light on RSV and other virus-associated mortality in the absence of hospital care. The Bill & Melinda Gates Foundation is also funding minimally invasive autopsies on children stillborn or dying, in both hospital and community settings, to better help define the causes of their deaths.

Despite the reduction in pneumonia—related childhood deaths in infants <5 years of age, less impact has been seen on pneumonia- and sepsis-related mortality among neonates [2, 3]. PERCH excluded neonates but may shed some light on the contribution of influenza, RSV, and pertussis to pneumonia etiology from 1 to 6 months of age, when maternal immunization strategies may reduce disease burden. The classic definition of pertussis was not included in the PERCH entry criteria, but cases presenting as severe pneumonia would have been included.

In view of the challenges, ascertaining the etiology of pneumonia, especially for commonly carried bacterial pathogens in children, is a daunting task. PERCH has approached this task with a thoughtful study design and state-of-the-art diagnostics. This study has achieved many important milestones including the standardization of methods in a complex multicountry study; the study paid careful attention to quality control; it delivered diagnostics through technology transfer to local laboratories; and it pulled together a unique collaboration of multiple investigators and collaborators. With the foundation set by the articles in this supplement, we look forward to the analysis of PERCH data in the future and its contribution to our understanding of both bacterial and viral etiologies of pneumonia.

## Notes

***Supplement sponsorship.*** This article appears as part of the supplement “Pneumonia Etiology Research for Child Health (PERCH): Foundational Basis for the Primary Etiology Results,” sponsored by a grant from the Bill & Melinda Gates Foundation to the PERCH study of Johns Hopkins Bloomberg School of Public Health, Baltimore, Maryland.

***Potential conflicts of interests.*** Both authors are employed by the Bill & Melinda Gates Foundation, which funded the PERCH study. Both authors have submitted the ICMJE Form for Disclosure of Potential Conflicts of Interest. Conflicts that the editors consider relevant to the content of the manuscript have been disclosed.
